# 35522 Implementing and Disseminating Translational Science Virtually, Successfully and Saving a Whole Lot of Money

**DOI:** 10.1017/cts.2021.541

**Published:** 2021-03-30

**Authors:** Christine Drury

**Affiliations:** Indiana University Clinical and Translational Sciences Institute

## Abstract

ABSTRACT IMPACT: We hosted the Indiana Clinical and Translational Sciences Annual meeting virtually this year which resulted in positive feedback survey scores over 90% and an estimated 87% cost savings OBJECTIVES/GOALS: COVID-19 has forced many in-person meetings to become virtual, not unlike our 2020 Indiana Clinical and Translational Sciences Institute Annual Meeting. However, where anecdotal feedback has shown dissatisfaction with some on-line meetings, we were able to exceed our goals of engaging our audience, securing positive feedback and even saving money. METHODS/STUDY POPULATION: More than 500 people attended the virtual 2020 Indiana Clinical and Translational Sciences Institute (CTSI) Annual Meeting on September 11. The event had two plenary speakers and was completely online, utilizing both Zoom and Microsoft Teams to connect participants with the presenters. Brian Druker, MD, director of the Knight Cancer Institute at Oregon Health & Science University, was the winner of this year’s August M. Watanabe Prize in Translational Research. He gave the first plenary presentation titled, ‘Imatinib as a Paradigm of Targeted Cancer Therapies.’ Consuelo Wilkins, MD, Vice President for Health Equity at Vanderbilt University Medical Center, gave the second plenary titled, ‘Confronting Racial Inequities through Research.' Concurrent online breakout rooms hosted the live poster session. RESULTS/ANTICIPATED RESULTS: Despite being conducted online, the virtual Indiana CTSI annual meeting registered more participants than in years past and secured high feedback scores of 90%, all while experiencing 87% cost savings over last year’s in-person meeting. By utilizing Microsoft Teams as a technology for attendees to the meeting to 'chat’ and 'network’ with one another during the poster presentations and virtual lunch break we were able to demonstrate the implementation of translational science through online plenary and general session presentations as well as the poster presentations. Mailing certificates to the poster winners in advance, allowed them to share their accolades with the audience by holding up their certificates once their winning posters were announced. An e-annual report also supported the success of the meeting. DISCUSSION/SIGNIFICANCE OF FINDINGS: The cost savings and traditionally high feedback scores received through this year’s Indiana CTSI annual meeting, mean virtual meetings are a viable way to disseminate and implement translational science. In addition the 2020 Indiana CTSI annual report received a Gold MarComm award, providing third party recognition of its impact.

